# Evaluation of Insecticides induced hormesis on the demographic parameters of *Myzus persicae* and expression changes of metabolic resistance detoxification genes

**DOI:** 10.1038/s41598-018-35076-1

**Published:** 2018-11-09

**Authors:** Muhammad Umair Sial, Zhenzhen Zhao, Lan Zhang, Yanning Zhang, Liangang Mao, Hongyun Jiang

**Affiliations:** State Key Laboratory for Biology of Plant Diseases and Insect Pests, Institute of Plant Protection, Chinese Academy of Agricultural Sciences, Beijing, 100193 P. R. China

## Abstract

Insecticide induced-hormesis is a bi-phasic phenomenon generally characterized by low-dose induction and high-dose inhibition. It has been linked to insect pest outbreaks and insecticide resistance, which have importance in the integrated pest management (IPM). In this paper, hormesis effects of four insecticides on demographic parameters and expression of genes associated with metabolic resistance were evaluated in a field collected population of the green peach aphid, *Myzus persicae* Sulzer. The bioassay results showed that imidacloprid was more toxic than acetamiprid, deltamethrin and lambda-cyhalothrin. After exposure to sublethal doses of acetamiprid and imidacloprid for four generations, significant prolonged nymphal duration and increased fecundity were observed. Subsequently, mean generation time (*T*) and gross reproductive rate (*GRR*) was significantly increased. Moreover, expression of *CYP6CY3* gene associated with resistance to neonicotinoids was increased significantly compared to the control. For pyrethriods, across generation exposure to sublethal doses of lambda cyhalothrin and deltamethrin prolonged the immature development duration. However, the expression of *E4* gene in *M. persicase* was decreased by deltamethrin exposure but increased by lambda cyhalothrin. Based on results, demographic fitness parameters were effected by hormetic dose and accompanied with detoxifying genes alteration, hence, which would be evaluated in developing optimized insect pest management strategies.

## Introduction

Pesticide induced hormesis is a biphasic phenomenon resulted by low dose stimulation and high dose inhibition following insecticides exposure^1,2^. Recently, insecticides induced-sublethal effects in various agricultural insect pests have been reported^3–5^. It has been shown that hormesis responses can accelerate insect population growth, result in insect pest resurgence^6–8^, and benefit resistance development^9–11^, which have significance for insect pest and insecticide resistant management^8^.

The green peach aphid, *Myzus persicae* (Sulzer) (Hemiptera: Aphididae) is an economically significant agricultural crop insect pest across the world that resulted in severe crop damages on over 400 plant species^12^. Therefore, numerous insecticides are used to control this insect and in turn, the resistance to multiple insecticides are developed^13^. The most common resistant mechanisms include target-site mutations and over-expression of detoxification enzymes^14,15^. In *M. persicae*, metabolic enzymes reported to confer resistance include esterase E4 (or the mediterranean variant FE4), giving broad-spectrum resistance to organophosphates, carbamates and pyrethroids, and cytochrome P450 CYP6CY3, conferring resistance to neonicotinoids^16^. Regarding to esterase mediated pyrethroid resistance in *M. persicae*, previous studies have indicated that esterase *E4/FE4* amplification was related to a certain resistance to deltermethrin^17^. In China, the field populations of *M. persicae* was detected to develop a high level of resistance to β-cypermethrin and cypermethrin, and high frequency of *FE4* amplification was significantly correlated with resistance of *M. persicae* to these two pyrethroid insecticides^18^. In the early 1990s, low level resistance to neonicotinoids was observed very soon after this class of insecticides was introduced to control *M. persicae*. Studies of Puinean *et al*. revealed that over-expression of a P450 gene, *CYP6CY3*, was related to neonicotinoid resistance^19^. Subsequently, the over-expression of *CYP6CY3* was identified in several field populations of *M. persicae*^16,18^.

Hormesis, an evolutionary adaptation of organisms response to environmental stress, has significance in insect pest management due to insects may be exposed to sublethal levels of pesticides. It has been documented that low dose exposure to insecticide can hasten the evolution of pesticide resistance by increasing mutation frequencies^20^. In *M. persicae*, hormesis effects induced by several insecticides have been reported^3–5,20–23^. In addition to stimulating responses on different insect’s life history traits, it is also reported that low dose insecticides exposure alters gene expression which has been associated with metamorphosis and reproductive development in *M. persicae*^22^. According to reports of Rix *et al*. exposure to hormetic concentrations of imidacloprid can prime offspring to better withstand subsequent insecticide stress, but not result in mutations in any of the examined nicotinic acetylcholine receptor subunits (nAChR) in a wild green-house population of *M. persicae*^20^.

In China, *M. persicae* is one of the most serious pests, which causes great economic losses every year. Recently, Tang *et al*. reported that the field populations of *M. persicae* collected from eleven sites including Langfang have developed multiple levels of resistance to permethrins and neonicotinoids, and over-expression of cytochorome P450 *CYP6CY3* and esterase *E4/FE4* genes was involved in resistance to neonicotinoids and pyrethroids, respectively^18^. Therefore, we studied the homesis effects on the demographic parameters of pyrethroid and neonicotinoid insecticides in *M. persicae* collected from greenhouse crops in Langfang. Moreover, we were interested to know whether hormetic responses may change the expression of cytochorome P450 *CYP6CY3* and esterase *E4/FE4*. These results may provide important information for developing optimized integrated pest resistant management strategies in *M. persicae*.

## Results

### Bioassays

The LC_50_ value was 381 mg L^−1^ (*df* = 5, *P* = 0.44) for deltamethrin and 1010 mg L^−1^ (*df* = 5, *P* = 0.47) for lambda cyhalothrin after 48 h exposure, respectively. Dose-response relationship data after 72 h exposure for LC_50_ to acetamiprid and imidacloprid was determined at 0.330 mg L^−1^ (*df* = 5, *P* = 0.5) and 0.089 mg L^−1^ (*df* = 5, *P* = 0.15), respectively (Table 1). Nonetheless, the LC_20_ values were selected to study homesis effects on demographic parameters and expression of cytochorome P450 *CYP6CY3* and esterase *E4/FE4* in *M. persicae* after multigenerational exposure to these four insecticides.

**Table 1:** Dose response toxicity assay of pyrethroids and neonicotinoids to adult *M. persicae*. ^a^Confidence limits; ^b^Standard error; ^c^Degrees of freedom; ^d^Chi-square value.

Insecticide Groups	Insecticides	LC_50_ (mg/l)	95%CL^a^	LC_20_ (mg/l)	95%CL^a^	Slope (±SE^b^)	*df* ^c^	*χ*2^d^	*P*
Pyrethroids	Deltamethrin	381	242–656	45.7	15.3–84.0	0.913 (±0.160)	5	4.74	0.448
	Lambda Cyhalothrin	1010	613–2030	96.7	29.0–184	0.825 (±0.157)	5	4.51	0.479
Neonicotinoids	Imidacloprid	0.089	0.048–0.155	0.024	0.007–0.046	1.49 (±0.190)	5	8.85	0.155
	Acetamiprid	0.330	0.230–0.490	0.065	0.033–0.102	1.19 (±0.170)	5	4.28	0.509

### Sublethal effects of neonicotinoids

Compared to the control group, the hormetic effects of two neonicotinoids, acetamiprid and imidacloprid on *M. persicae* were assessed after exposure to the sublethal concentrations for one and four generations, respectively. As shown in Table 2, after exposure to the sublethal concentration (LC_20_) of acetamiprid for one generation, the development time of 4th instar (N4), female longevity and total fecundity were significantly increased among all tested biological parameters of *M. persacae*, comparing with the control group. After exposure four generations, the duration time of nymph stages (N1, N2, N3) and pre-adult, female longevity, total fecundity and the total preoviposition period (TPOP) was significantly enhanced, respectively. In case of imidacloprid, female longevity, the per day fecundity and total fecundity of *M. persicae* were increased significantly compared to the control group. Under continuous treatment to sublethal dose of imidacloprid for four generations, the nymphal developmental duration of the third instar and the female longevity, per day fecundity and total fecundity were significantly prolonged, and the total fecundity was significantly increased.

**Table 2:** Developmental time length, longevity, APOP, TPOP, fecundity of F1 and F4 generations of *M. persicae* following sublethal neonicotinoids doses.

Stage durations	Control	Acetamiprid		Imidacloprid		*df*	*F* value	*P* value
		F1	F4	F1	F4			
1^st^ Instar (N1)	1.06 ± 0.03	1.21 ± 0.06	1.61 ± 0.07*	1.16 ± 0.06	1.18 ± 0.06	4	14.0	<0.001
2^nd^ Instar (N2)	1.47 ± 0.09	1.09 ± 0.04	1.80 ± 0.08*	1.43 ± 0.08	1.32 ± 0.10	4	10.6	<0.001
3^rd^ Instar (N3)	1.33 ± 0.07	1.43 ± 0.11	1.90 ± 0.09*	1.49 ± 0.11	1.82 ± 0.13*	4	6.17	<0.001
4^th^ Instar (N4)	1.72 ± 0.10	1.94 ± 0.13*	1.50 ± 0.08	1.40 ± 0.11	1.62 ± 0.10	4	3.25	0.013
Preadult	5.53 ± 0.10	5.66 ± 0.13	6.95 ± 0.12*	5.47 ± 0.12	5.94 ± 0.13	4	26.4	<0.001
Longevity	14.0 ± 0.14	14.8 ± 0.13*	15.0 ± 0.11*	15.0 ± 0.16*	16.0 ± 0.15*	4	27.1	<0.001
Fecundity/day	2.95 ± 0.04	3.04 ± 0.05	3.12 ± 0.05	3.22 ± 0.04*	3.18 ± 0.04*	4	4.10	0.003
Fecundity	41.1 ± 0.61	44.9 ± 0.64*	46.3 ± 0.72*	48.1 ± 0.57*	50.9 ± 0.62*	4	27.1	<0.001
APOP^a^	1.09 ± 0.04	1.29 ± 0.10	1.27 ± 0.07	1.37 ± 0.09	1.41 ± 0.08	4	0.61	0.611
TPOP^b^	6.63 ± 0.10	6.89 ± 0.19	8.27 ± 0.14*	6.86 ± 0.16	7.35 ± 0.17	4	16.3	<0.001

Data presented in table are mean ± SE, while the symbol (*) is used to denote significant difference (*P* < 0.05). ^a^Adult pre-oviposition period; ^b^Total pre-oviposition period.

Sublethal effects of neonicotinoids on the demoghraphic parameters was calculated and shown in Table 3. Intrinsic rate of increase (*r*_*m*_) and finite rate of increase (*λ*) in *M. persicae* exposed to acetamiprid and imidacloprid were reduced significantly compared to the control group. Likewise, mean generation time (*T*) and gross reproductive rate (*GRR*) in the sublethal exposure of both neonicotinoids group was higher than that of the control group. While, there was no significant difference in the net reproductive rate (*R*_0_) between *M. persicae* exposed to two neonicotinoids and the control group.

**Table 3:** Demographic parameters of of F1 and F4 generations of *M. persicae* following exposure to sublethal dose of neonicotinoids.

Parameters	Original					Bootstrap (±SE)				
	Control	Acetamiprid		Imidacloprid		Control	Acetamiprid		Imidacloprid	
		F1	F4	F1	F4		F1	F4	F1	F4
*r* (/day)^a^	0.304	0.284	0.263	0.287	0.275	0.303 ± 0.006	0.284 ± 0.010	0.263 ± 0.006*	0.286 ± 0.009	0.275 ± 0.009*
*λ* (/day)^b^	1.35	1.33	1.30	1.33	1.32	1.35 ± 0.008	1.33 ± 0.013	1.30 ± 0.008*	1.33 ± 0.012	1.32 ± 0.012*
*T* (days)^c^	11.7	12.1	13.8	12.2	12.8	11.7 ± 0.123	12.1 ± 0.227	13.8 ± 0.160*	12.2 ± 0.166*	12.8 ± 0.161*
*Ro* (offspring/individual)^d^	35.4	31.4	38.0	33.6	34.6	35.3 ± 2.06	31.4 ± 2.96	38.0 ± 2.58	33.6 ± 3.15	34.6 ± 3.39
GRR (offspring/individual)^e^	43.0	45.7	47.7	49.1	51.0	43.0 ± 0.78	45.7 ± 0.829*	47.7 ± 0.809*	49.0 ± 0.909*	51.0 ± 0.763*

Data presented in table are mean ± SE, while the symbol (*) is used to denote significant difference (*P* < 0.05). ^a^Intrinsic rate of increase; ^b^Finite rate of increase; ^c^Generation time; ^d^Net reproductive rate; ^e^Gross reproductive rate.

### Sublethal effects of pyrethroids

As shown in Table 4, all tested biological parameters of *M. persacae* after exposure to the sublethal concentration (LC_20_) of deltamethrin for one generation were not affected significantly except longevity. Regarding for exposure four generations, the duration of nymphal stages (N3 and N4) and pre-adult, and the adult preoviposition period (TPOP) was significantly prolonged, respectively. The developmental time of 1st, 3rd instar nymphs, daily fecundity, adult preoviposition period (APOP) and total preoviposition period (TPOP) was significantly increased compared to the control group after exposure to the sublethal concentration (LC_20_) of lambda cyhalothrin for four generations. When exposure to the sublethal concentration of deltamethrin was continued to F4 generation, the 1^st^ instar nymph, pre-adult and the adult preoviposition period (TPOP) was significantly increased.

**Table 4:** Developmental time length, longevity, APOP, TPOP, fecundity of F1 and F4 generations of *M. persicae* following sublethal pyrethroids doses.

Stage durations	Control	Deltamethrin		Lambda Cyhalothrin		*df*	*F* value	*P* value
		F1	F4	F1	F4			
1^st^ Instar (N1)	1.06 ± 0.03	1.19 ± 0.06	1.25 ± 0.06	1.31 ± 0.07*	1.82 ± 0.09*	4	19.3	<0.001
2^nd^ Instar (N2)	1.47 ± 0.09	1.48 ± 0.10	1.47 ± 0.09	1.49 ± 0.07	1.26 ± 0.06	4	1.12	0.349
3^rd^ Instar (N3)	1.33 ± 0.07	1.64 ± 0.08	1.84 ± 0.09*	1.78 ± 0.11*	1.60 ± 0.10	4	4.80	0.001
4^th^ Instar (N4)	1.72 ± 0.10	1.50 ± 0.09	1.98 ± 0.10*	1.32 ± 0.07	1.29 ± 0.07	4	10.4	<0.001
Preadult	5.53 ± 0.10	5.81 ± 0.08	6.53 ± 0.08*	5.90 ± 0.13	5.98 ± 0.09*	4	13.5	<0.001
Longevity	14.0 ± 0.14	14.8 ± 0.13*	14.4 ± 0.15	14.2 ± 0.20	14.4 ± 0.18	4	3.90	0.004
Fecundity/day	2.95 ± 0.04	2.75 ± 0.04	2.94 ± 0.04	3.02 ± 0.04*	2.87 ± 0.04	4	4.98	<0.001
Fecundity	41.1 ± 0.61	40.8 ± 0.62	42.0 ± 0.60	42.6 ± 0.63	41.2 ± 0.62	4	1.40	0.234
APOP^a^	1.09 ± 0.04	1.10 ± 0.04	1.00 ± 0.00?	1.17 ± 0.05*	1.07 ± 0.04	4	2.11	0.081
TPOP^b^	6.63 ±± 0.10	6.90 ± 0.10	7.53 ± 0.08*	7.07 ± 0.13*	7.05 ± 0.11*	4	9.58	<0.001

Data presented in table are mean ± SE, while the symbol (*) is used to denote significant difference (*P* < 0.05). ^a^Adult pre-oviposition period; ^b^Total pre-oviposition period.

Sublethal effects of pyrethroids on the demoghraphic parameters was shown in Table 5. Intrinsic rate of increase (*r*_*m*_) and finite rate of increase (*λ*) to both insecticides were reduced significantly compared to the control group. Likewise, mean generation time (*T*) was higher both at F1 and F4 generations than that of the control group. The gross reproductive rate (*GRR*) was increased significantly at F1 generation.

**Table 5:** Demographic parameters of F1 and F4 generations of *M. persicae* following exposure to sublethal dose of pyrethroids.

Parameters	Original					Bootstrap (±SE)				
	Control	Deltamethrin		Lambda Cyhalothrin		Control	Deltamethrin		Lambda Cyhalothrin	
		F1	F4	F1	F4		F1	F4	F1	F4
*r* (/day)^a^	0.304	0.291	0.278	0.287	0.285	0.303 ± 0.006	0.291 ± 0.006	0.278 ± 0.004*	0.286 ± 0.007	0.285 ± 0.006*
*λ* (/day)^b^	1.35	1.34	1.32	1.33	1.33	1.35 ± 0.008	1.34 ± 0.008	1.32 ± 0.006*	1.33 ± 0.009	1.33 ± 0.008*
*T* (days)^c^	11.7	12.1	13.0	12.4	12.4	11.7 ± 0.123	12.1 ± 0.116*	13.0 ± 0.120*	12.4 ± 0.162*	12.4 ± 0.138*
*Ro* (offspring/individual)^d^	35.4	34.3	37.8	35.0	34.6	35.3 ± 2.06	34.3 ± 2.19	37.7 ± 1.85	35.0 ± 2.37	34.6 ± 2.20
GRR (offspring/individual)^e^	43.0	41.7	43.0	47.0	44.0	43.0 ± 0.78	41.7 ± 0.757	43.0 ± 0.564	47.0 ± 1.13*	44.0 ± 0.929

Data presented in table are mean ± SE, while the symbol (*) is used to denote significant difference (*P* < 0.05). ^a^Intrinsic rate of increase; ^b^Finite rate of increase; ^c^Generation time; ^d^Net reproductive rate; ^e^Gross reproductive rate.

### Insecticides resistance linked genes expression

**Figure 1:**
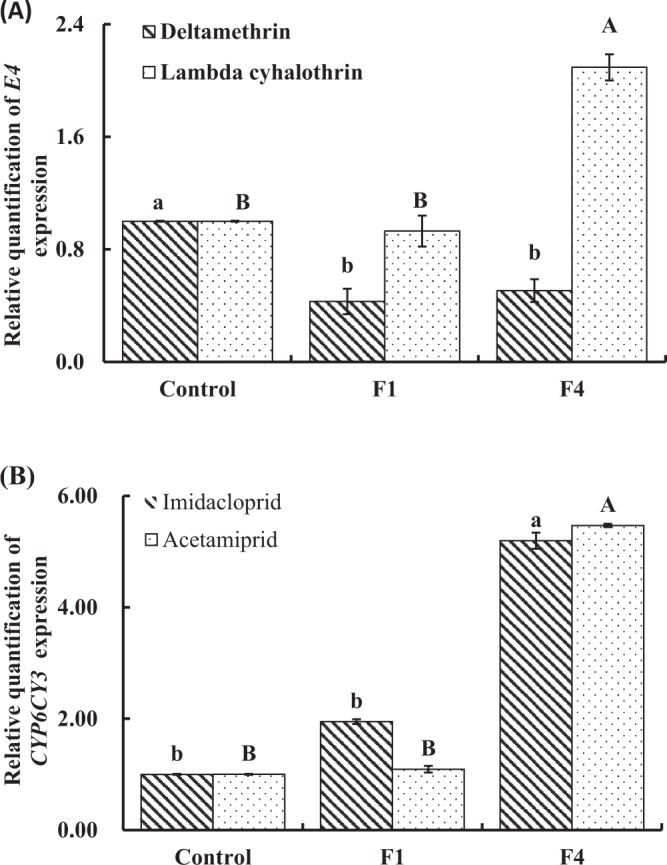
Relative expression of cytochrome esterase *E4* (**A**) and P450 *CYP6CY3* (**B**) genes (±SE) in *M. persicae* at initial generation (control), F1 and F4 generation (neonicotinoid and pyrethroid exposure for one and four generations, respectively). Bars having different letters are significantly different, respectively (*P* < 0.05). Lower case (a,b) and uppercase (**A**,**B**) letters are used to distinguish the two insecticides of one class in (**A**) or (**B**).

In *M. persicae*, resistance to neonicotinoid and to permethrin insecticides has been associated with over-expression of cytochrome P450 *CYP6CY3* and esterase *E4/EF4* genes, respectively. In this paper, the hormetic effects of sublethal dose of insecticides on cytochrome P450 *CYP6CY3* and esterase *E4* genes were detected (Fig. 1). After exposure to imidacloprid for one and four generations, cytochrome P450 *CYP6CY3* gene expression was increased to 1.95- and 5.20-fold. In the case of acetamiprid, there was 1.09-fold and 5.47-fold increased expression in P450 *CYP6CY3* gene at F1 and F4, respectively. Interestingly, exposure to deltamethrin for one and four generations, expression of *E4*-esterase gene was decreased to 0.43- and 0.51-fold, respectively. *E4*-esterase gene expression was 2.09-fold of that in the control group after exposure to lambda cyhalothrin for four generations in *M. persicae*, but only 0.93-fold at F1.

## Discussion

Adaptive mechanism attitude has been chosen by many organisms for their fetus survival and reproduction in stressful surroundings. In the study, we have shown the short and prolonged sublethal neonicotinoid and pyrethroid insecticides exposure to *M. persicae* significantly resulted in increased reproduction across different generations. We also have disclosed that insecticides hormetic exposure could significantly increase the expression of detoxifying genes involved in insecticide resistance. All these results suggested that hormesis was involved in *M. persicae* adaptive mechanisms followed by sublethal exposure of neonicotinoid and pyrethroid insecticides^24^.

In our study, the nymphal instar period (N1, N2, N3) was significantly prolonged after the sublethal exposure of acetamiprid, while only third instar showed prolonged time period in case of imidacloprid exposure. The level of female longevity, per day fecundity and total fecundity was significantly increased after the sublethal exposure of neonicotinoids both on F1 and F4 generations. Previously, the effects of insecticide induced hormesis in *M. persicae* have been studied across multiple generations^3–5,20–23^, and also reported in other aphid species^25^, leafhopper species and citrus plant thrips^26–29^. Similarly, the increased population and survival of *M. persicae* by the exposure of sublethal concentrations of imidacloprid, azadirachtin and azinphosmethyl, have been reported^30–33^. Moreover, extended time length for the development of *A. glycines* Matsumura, *Brevicoryne brassicae* and *A. gossypii* to the low doses of imidacloprid confirmed our outcomes^34–39^. The phenomenon of extended development after low doses exposure could be linked that exposed aphids needed long-term nutrients enrichment and mass reproduction to cope with chemical or any stressor.

Low doses of pyrethroids application have also shown effects on the developmental durations of insects as reported by Kerns and Stewart^37^. In current study, sublethal exposure of pyrethroids on *M. persicae* for one and four generations delayed only the development of nymphal instars. There was no significant difference in reproduction between the control and low dose exposure groups. Same lack of stimulatory effects has been shown in *A. gossypii* when exposed to sulfoxaflor and imidacloprid with LC_20_^35,38^.

In our experiments, the intrinsic rate (*r*_*m*_) and finite rate (λ) both were reduced as compared to control group and it was supported by studies of Zeng *et al*.^5^, Wang *et al*.^23^, and Tang *et al*.^4^. By comparing the demographic parameters of *M. persicae*, it was shown that the population was effected with different treatments. For evaluation of insecticides effects, it is recommended to study the insect life parameters. Anyhow, the increased generation time (*T*) and gross reproductive rate (*GRR*) may suggest that sublethal concentrations, at some extant, could suppress or slow the growth of *M. persicae*, and these outcomes have also been reported in *Bradysia odoriphaga* and *Hippodamia variegate*^39,40^.

In this paper, the expression of esterase *E4* and P450 *CYP6CY3* genes was changed in *M. persicae* following low dose exposure to pyrethroids and neonicotinoids. Former studies have demonstrated that sublethal concentrations of insecticides or hormetic doses are involved in the alteration of detoxifying genes expression. Ayyanath *et al*.^3,22^ have quantified the expression of different genes, including *Hsp*, *FPPS I*, *OSD*, *ANT* and *TOL* following insecticides low doses exposure on *M. persicae*. In present study, esterase *E4* gene was increased about 2.09-fold after lambda cyhalothrin low dose exposure. However, the relative expression levels of *E4* mRNA was inhibited after exposure to deltamethrin, which might be considered as fitness costs to another positive adaptive mechanisms. It has been alleged that hormesis could lead an adaptive mechanism resulted to promote organisms phenotypic plasticity that cope ongoing and deleterious environmental variations. Our results suggested that *M. persicae* developed different adaptive pattern to low dose exposure of lambda cyhalothrin and deltamethrin. The constitutional and stereo-chemical structure of pyrethroids played an important role during the continuous exposure to pyrethroids in insects^41,42^. Additionally, during the deltamethrin exposure in insects, different mechanisms have been documented, including the elevation of carboxylesterase activity through gene over-expression, point mutations within the carboxylesterase genes which change their substrate specificity, and point mutations in sodium channel^42,43^.

P450-*CYP6CY3* gene expression was increased in *M. persicae* following imidacloprid and acetamiprid low dose exposure at F4. Many studies have shown that the increased expression of *CYP6CY3* is associated with increased metabolism which leads to insecticide resistance^19,44,45^. It was expected that insecticides hormetic dose exposure on insect would result in frequent alteration or expression of detoxifying genes. In this paper, exposure to imidacloprid and acetamiprid for four generations induced obviously the over-expression of P450-*CYP6CY3*, suggesting that hormetic exposure could significantly increase the expression of detoxifying genes involved in *M. persicae* resistance to pesticides. Therefore, *M. persicae* resistance to neonicotinoids might be hasten by low dose exposure to this class of insecticides, which would be evaluated in developing optimized insect pest management strategies.

In conclusion, continuous asymmetrical application and degradation of insecticides in fields resulted that frequently insects are exposed to hormetic concentrations of insecticides. Two pyrethroids and neonicotinoids, as commonly used to control aphids, were used with low doses over four generations to study *M. persicae* demographic parameters and as well as their impact on gene expression. In our toxicity bioassay, the imidcloprid was more toxic as compared to other insecticides. Based on the present study, the developmental stages were delayed by the exposure of hormetic doses and potentially increased the *M. persicae* reproduction. Anyhow, the potential effects of sublethal doses should be evaluated on natural enemies in a long term way, also to control aphid-borne viruses and potentially application in IPM under field conditions. Previously, it has been concluded that xenobiotics could enhance the production of detoxifying genes and resulted in resistance. The shown study is the only one to disclose the genes induction expression following pyrethroids and neonicotinoids low doses exposure across over four generations, coinciding with upsurges of population reproduction.

## Materials and Methods

### Ethics statement

Neither permission was required for insect collection, no species used in the study were endangered or protected.

### Insects rearing

*M. persicae* population was collected from experimental Lang fang Station of the Chinese Academy of Agricultural Sciences (CAAS), Hebei, China during April 2017. Aphids were reared and maintained on Chinese cabbage leaves under the climatic chamber without any insecticides exposure. The temperature in climatic chamber was maintained at 25 ± 2 °C and 65 ± 5 RH, whilst light day photoperiod was 16:8 h.

### Insecticides preparation

Four insecticides were used in this study, including two pyrethroids (deltamethrin, 95.3% and lambda-cyhalothrin, 96%) offered by Guangxi Tianyuan Chemical Co., Ltd., and two neonicotinoids (acetamiprid, 97.5% and imidacloprid, 96%) offered by Shandong Lianhe Chemical Co., Ltd. Each insecticide was suspended in distilled water by using <1% ethyl alcohol (EtOH) to get a stock solution. Different concentrations of deltamethrin (2000, 1000, 500, 250, 125, 62.5 and 31.25 mg L^−1^), lambda-cyhalothrin (4000, 2000, 1000, 500, 250, 125 and 62.5 mg L^−1^), acetamiprid (2, 1, 0.5, 0.25, 0.125, 0.0625 and 0.03125 mg L^−1^) and imidacloprid (1, 0.5, 0.25, 0.125, 0.0625, 0.03125 and 0.015625 mg L^−1^) were prepared. Insecticides dilutions were immediately used to prevent any chemical decomposition.

### Leaf-Dip Toxicity Bioassay

The leaf dip method was used to test the toxicity of mentioned insecticides^5,18^. Briefly, cabbage leaf discs were individually dipped into insecticide solution for about 20–30 s and air-dried at room temperature for 1–2 h. For the control group, leaves were treated with distilled water containing <1% EtOH. The cabbage discs were then kept by turning their upside down onto petri dishes. The petri dishes bottom was contained 1% agar solution for humidity control and 15 apterous young individuals were inoculated. Three petri dishes were conducted for each concentration. The Parafilm (Fisher Scientif, Ottawa, Canada) was used to seal the petri dishes to prevent insect escape. All petri dishes were kept in climatic chamber under controlled temperature 25 ± 2 °C, relative humidity 65 ± 5% and light day photoperiod was 16:8 h. The mortality was assessed after 48 h for pyrethroids and 72 h for neonicotinoids. Each bioassay was repeated three times. The dose-response for each insecticide was assessed by using probit analysis in SPSS 22.0 version.

### Sublethal dose exposure

The sublethal dose effects (LC_20_) on *M. persicae* development were assessed for consecutive over four generations. The leaf discs were dipped in insecticides solution (LC_20_) or control (water treated) and placed in a petri dishes as described above. The adult apterous aphids were released onto treated and control petri dishes. After 24 h exposure, 50 the first instars nymphs (F1) from the treated or control group were randomly selected and individually placed in a separate petri dish to study life parameters^5,18^. Whilst, remaining neonates (F1) were reared for further generations and the leaf discs inside petri dishes were replaced every 2–3 days for both the treated and control group. At the fourth generation (F4), the same method was repeated again to study life parameters with the first instars. During the experiments, the petri dishes were placed inside climatic chamber under the controlled conditions as described above.

### Demographic Analysis

The raw data, daily survival of new born nymphs and adults, and their longevity were used to calculate following demographic parameters by using the TWOSEX-MSChart 2015.045^46^. Net reproductive rate: R_0_ = ∑ *l*_*x*_*m*_*x*,_ the number of times in which an individual population will get multiply per generation; Generation time: T = (∑*l*_*x*_*m*_*x*_)/R_0_, the average time length that separate a female birth of one generation from the next; Intrinsic rate of increase: *r*_*m*_ = *l*_*n*_(R_0_)/T, species innate capacity that increase in numbers; Gross reproductive rate: G = ∑*m*_*x*_, the average number of females produced; Finite rate of increase: λ = *e*^*r*^_*m*_, the rate in which a population get multiply in one day. Whereas, *x* is the individual pivotal age in days, *l*_*X*_ and *m*_*x*_ are the percentage of age specific survival and fecundity at given age *x*, accordingly.

### Total RNA Extraction and cDNA Synthesis

Total 20–30 aphids from each treatment were pooled as one biological replicate to isolate RNA with RNeasy^®^ mini kits (Qiagen, ON, Canada). The quantitative and qualitative analysis (A260/280 > 2.0) for RNA was determined by Nanodrop ND-1000 (NanoDrop Technologies, Wilmington, DE), and also checked through gel electrophoresis analysis (1% gel). The cDNA was then synthesized using Omniscript^®^ Reverse Transcript Kit (Qiagen, ON, Canada) by a microgram of total RNA and stored at −20 °C for later analysis.

### Gene expression

Quantitative real-time PCR (qRT-PCR) was analyzed using *TransStart*^®^ Top Green qPCR SuperMix (Transgen, Beijing, China) and performed on CFX ConnectTM Real-Time System (BIO-RAD, Singapore). Primers for esterases-*E4* (GenBank accession no. X74554) and P450 cytochrome *CYP6CY3* (GenBank accession no. HM009309) were listed in Table 6. The *β*-actin was used as an internal reference gene. The reaction was performed in 50 µL tube according to manufacturer directions of total 20 µL reaction mixture. The reaction contained of a 10 µL of 2 × *TransStart*^®^ Top Green qPCR SuperMix, 0.4 µL of each forward and reverse primer, ddH_2_O 8.2 µL and 1 µL template. The cyclic thermal procedure involved an initial denaturation step at 94 °C for 30 s, following 40 cycles at 94 °C for 30 s for 5 s and 60 °C for 30 s and then a dissociation step was performed. The qRT-PCR analysis was included three independent biological replicates for each treatment. The fold change of target genes was calculated using the relative quantitative method (2^−ΔΔCt^) described by Pfaffl^47^.

**Table 6:** Primers used in real-time PCR. *F*, forward primer; *R*, reverse primer; *bp*, base pairs.

Gene	Primers sequence (5′-3′)		Amplicon size (bp)	Reference
*CYP6CY3*	F	CGGGGTGACGATCATCTATT	128	Puinean *et al*.^19^
	R	GGGTGGTCTTTTGACAAAGC		
*E4*	F	AAACTTTCCTTTTACACCGTT	160	Puinean *et al*.^19^
	R	TCTAAGCCAAGAAATGTTGAAA		
*β-actin*	F	GGTGTCTCACACACAGTGCC	120	Puinean *et al*.^19^
	R	CGGCGGTGGTGGTGAAGCTG		

### Statistical analysis

Data were statistically analyzed using one-way analysis of variance (ANOVA) followed by least significant difference (LSD) test in SPSS software version 22.0. Results were considered significant when *P* was < 0.05. For precise estimation, the bootstrap method was designed for *M. persicae* demographic parameters with 100000 replications and mean values, as well as standard error were calculated in TWOSEX-MSChart 2015.045^46^. For the expression of relative quantities of gene *P450-CYP6CY3* and *E4-esterase*, Tukey comparison were used to test mean differences among treatments across generations and separated by LSD tests.
